# Association Between Body Mass Index, Obesity, and Clinical Outcomes Following Coronary Artery Bypass Grafting in Brazil: An Analysis of One Year of Follow-up of BYPASS Registry Patients

**DOI:** 10.21470/1678-9741-2023-0133

**Published:** 2024-02-26

**Authors:** Rodrigo Santin Ramos, Isadora Salvador Rocco, Marcela Viceconte, José Amalth do Espírito Santo, Otavio Berwanger, Renato Hideo Nakagawa Santos, Renato Abdala Karam Kalil, Fabio B. Jatene, Alexandre Biasi Cavalcanti, Alexandre Cabral Zilli, Walace de Souza Pimentel, Nelson Américo Hossne Junior, João Nelson Rodrigues Branco, Renata Trimer, Paulo Roberto Barbora Evora, Walter J. Gomes, Solange Guizilin, the BYPASS Registry Study Group.

**Affiliations:** 1 Cardiology Postgraduate Program, Escola Paulista de Medicina, Universidade Federal de São Paulo, São Paulo, São Paulo, Brazil; 2 Cardiovascular Surgery Discipline, Escola Paulista de Medicina, Universidade Federal de São Paulo, São Paulo, São Paulo, Brazil; 3 Instituto de Pesquisa - IP, Hospital do Coração - HCor, São Paulo, São Paulo, Brazil; 4 Instituto de Cardiologia do Rio Grande do Sul, Fundação Universitária de Cardiologia, Porto Alegre, Rio Grande do Sul, Brazil; 5 Cardiovascular Surgery Division, Instituto do Coração - InCor, Hospital das Clínicas da Faculdade de Medicina da Universidade de São Paulo - HCFMUSP, São Paulo, São Paulo, Brazil; 6 Department of Physical Therapy, Universidade Federal de São Carlos, São Carlos, São Paulo, Brazil; 7 Department of Surgery and Anatomy, Escola de Medicina de Riberão Preto, Universidade de São Paulo, Ribeirão Preto, São Paulo, Brazil

**Keywords:** Body Mass Index, Obesity Paradox, Mortality, Coronary Artery Bypass, Postoperative Complications, Registries

## Abstract

**Objective:**

To investigate the association between body mass index (BMI), obesity, clinical outcomes, and mortality following coronary artery bypass grafting (CABG) in Brazil using a large sample with one year of follow-up from the Brazilian Registry of Cardiovascular Surgeries in Adults (or BYPASS) Registry database.

**Methods:**

A multicenter cohort-study enrolled 2,589 patients submitted to isolated CABG and divided them into normal weight (BMI 20.0-24.9 kg/m^2^), overweight (BMI 25.0-29.9 kg/m^2^), and obesity (BMI > 30.0 kg/m^2^) groups. Inpatient postoperative outcomes included the most frequently described complications and events. Collected post-discharge outcomes included rehospitalization and mortality rates within 30 days, six months, and one year of follow-up.

**Results:**

Sternal wound infections (SWI) rate was higher in obese compared to normal-weight patients (relative risk [RR]=5.89, 95% confidence interval [CI]=2.37-17.82; *P*=0.001). Rehospitalization rates in six months after discharge were higher in obesity and overweight groups than in normal weight group (χ^2^=6.03, *P*=0.049); obese patients presented a 2.2-fold increase in the risk for rehospitalization within six months compared to normal-weight patients (RR=2.16, 95% CI=1.17-4.09; *P*=0.045). Postoperative complications and mortality rates did not differ among groups during time periods.

**Conclusion:**

Obesity increased the risk for SWI, leading to higher rehospitalization rates and need for surgical interventions within six months following CABG. Age, female sex, and diabetes were associated with a higher risk of mortality. The obesity paradox remains controversial since BMI may not be sufficient to assess postoperative risk in light of more complex and dynamic evaluations of body composition and physical fitness.

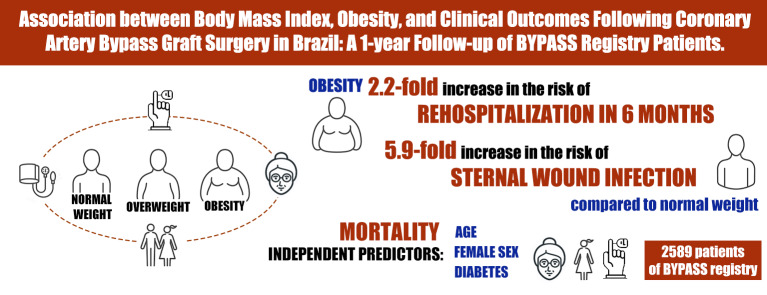



## INTRODUCTION

**Table:** 

Abbreviations, Acronyms & Symbols			
**ARDS**	**= Acute respiratory distress syndrome**	**ICU**	**= Intensive care unit**
**BMI**	**= Body mass index**	**LCOS**	**= Low cardiac output syndrome**
**BYPASS**	**= Brazilian Registry of Cardiovascular Surgeries in Adults**	**LVEF**	**= Left ventricular ejection fraction**
**CABG**	**= Coronary artery bypass grafting**	**MI**	**= Myocardial infarction**
**CAD**	**= Coronary artery disease**	**MV**	**= Mechanical ventilation**
**CI**	**= Confidence interval**	**PCI**	**= Percutaneous coronary intervention**
**COPD**	**= Chronic obstructive pulmonary disease**	**RBC**	**= Red blood cells**
**CPB**	**= Cardiopulmonary bypass**	**RR**	**= Relative risk**
**CVD**	**= Cardiovascular disease**	**STS**	**= Society of Thoracic Surgeons**
**EuroSCORE**	**= European System for Cardiac Operative Risk Evaluation**	**SWI**	**= Sternal wound infections**
**GzLM**	**= Generalized linear model**		

The vertiginous growth of obesity rates in the Brazilian population has concerned public health organizations due to the negative impact on the economy with the high cost of medical expenses. The average expenses for cardiovascular diseases alone in the obese population are estimated around R$2.5 billion (roughly 480 million US dollars) a year in the Sistema Único de Saúde (Brazilian unified national health system). According to a recent report, the rate of use of health care services in Brazil increases substantially in obese and overweight individuals, compared to the eutrophic population^[1]^.

Therefore, it becomes essential to identify the profile of patients with cardiac disease to delineate appropriate strategies for public health promotion and allocation of resources for cardiac surgery. Following the establishment of the Society of Thoracic Surgeons (STS) Adult Cardiac Surgery Database in 1989, many other national and continental databases were instituted to gather information on current trends and to improve the quality assessment and advance of cardiovascular surgery in their respective areas^[2]^. An important headway was provided with the inception of the first Brazilian national database of cardiovascular surgery in adults, the Brazilian Registry of Cardiovascular Surgeries in Adults (BYPASS) Registry^[3-5]^.

Among the most common comorbidities presented by obese people are cardiovascular diseases, such as coronary artery disease (CAD), high blood pressure, heart failure, and others. Coronary artery bypass grafting (CABG) is a proven efficient treatment option for these individuals, reducing risks associated with myocardial infarction and death, besides alleviating angina symptoms. An *obesity paradox* has been described, which reflects a relationship between obesity and reduced mortality, compared with normal weight. It refers to counter-intuitive epidemiological evidence suggesting improved health outcomes for obese individuals in a variety of clinical situations^[6,7]^. This paradoxical association has been demonstrated in diabetes, end-stage renal disease, hypertension, heart failure, established CAD, and peripheral arterial disease^[8-10]^. Studies examining the association between obesity and adverse outcomes following cardiac surgery have reported conflicting results^[11-16]^. Obesity may affect CABG patients in an advantageous or neutral manner, but are at odds with prior studies which suggest a higher mortality and morbidity in obese patients compared with normal-weight patients following CABG^[14,15,17]^.

However, most of these studies had a short-term follow-up and have not been explored in developing countries, where there are different patterns of socioeconomic status-related obesity. We designed the current study to investigate the association between body mass index (BMI), obesity, clinical outcomes, and mortality following cardiac surgery, with one year of follow-up, using information from the BYPASS Registry database. We sought to determine if BMI, and particularly obesity, is a predictor in determining outcomes following CABG.

## METHODS

This multicenter, observational cohort study uses data from the BYPASS Registry database. The BYPASS project is a national heart surgery registry, owned and funded by the Sociedade Brasileira de Cirurgia Cardiovascular (or SBCCV).

The participation of cardiovascular surgery centers in the BYPASS project was voluntarily convened and involved institutions located across the whole Brazilian territory. The 17 participating centers are well distributed among the following regions of the country: Southeast (n=8), Northeast (n=5), South (n=3), and Midwest (n=1). Informed consent form was signed by each patient following the national standards of clinical research already approved by the ethics and research committee of the coordinating center and each participating institution. All participating institutions were requested to complete the structured questionnaire, pertaining to the entire performed procedures and the related outcomes.

### Study Population

Adult patients over 18 years of age submitted to isolated CABG were prospectively included in the current analysis. Patients who refused to sign the informed consent or had chronic obstructive pulmonary disease (COPD), previous cardiac surgery, or end-stage renal disease were excluded.

Baseline characteristics on index date included age, sex, and BMI. BMI was calculated as weight (kg)/height (m^2^), and patients were divided into *normal weight* (BMI 20.0 to 24.9 kg/m^2^), *overweight* (BMI 25.0 to 29.9 kg/m^2^), and *obesity* (BMI > 30.0 kg/m^2^) groups, based on the World Health Organization classification (or WHO) ^[18]^. The following comorbidities were assessed: diabetes, smoking history (current, ex-smoking, and never smoked), peripheral vascular disease, cerebrovascular disease, congestive heart failure, hypertension, elective or emergency surgery, and left ventricular ejection fraction.

### Clinical Outcomes

Postoperative clinical outcomes during the inpatient period included the most frequently reported complications and events according to the STS guidelines (stroke, arrhythmia, cardiogenic shock, low cardiac output syndrome, major bleeding [a drop in hemoglobin of at least 3.0 g/dL or requiring transfusion of two or more units of whole blood/packed red blood cells] or causing hospitalization, permanent injury or need for surgery, blood transfusion, acute renal failure [serum creatinine ≥ 2.0 mg/ day and anuria for 12 hours or urine output < 0.3 mL/kg/hour for six consecutive hours], sternal wound infection (SWI), and prolonged mechanical ventilation (MV) (> 24 hours). The duration of postoperative hospital stay was recorded for all patients. A prolonged intensive care unit (ICU) stay was defined as ≥ 5 days and prolonged hospitalization as ≥ 11 days^[19,20]^.

Post-discharge outcomes collected included rehospitalization rates within 30 days, six months, and one year of discharge, and mortality at 30 days, six months, and one year of follow-up.

### Statistical Analysis

The numeric data were described by mean ± standard deviation, in the presence of normal distribution, otherwise as median and interquartile range. The categorical data were presented by absolute frequencies (n) and relative frequencies (%). To explore clinical and anthropometric data among groups, the one-way analysis of variance for independent samples was used to compare normally distributed data, the Kruskal-Wallis test was performed to discrete and non-Gaussian data, and the χ^^2^^ test was used to compare categorical data among groups.

Generalized linear model (GzLM) was used to explore the association of postoperative outcomes with grouping and clinical variables. A logistic distribution was adopted due to the binary nature of dependent variables: rehospitalization, need for surgical intervention, postoperative complications, and mortality. Holm post hoc test was used to investigate pairwise comparisons. Statistical analysis was performed using the statistical software Jamovi (2.3.21). A α < 0.05 was used to consider statistical level of significance.

## RESULTS

**Fig. 1:**
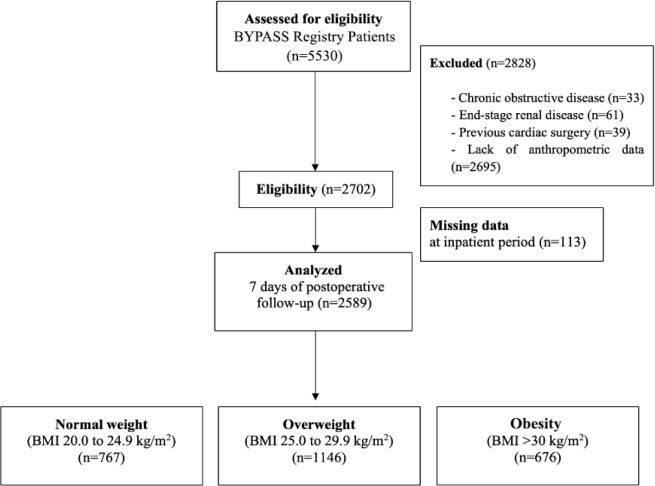
Flowchart of consecutive patients enrolled in the study. BMI=body mass index; BYPASS=Brazilian Registry of Cardiovascular Surgeries in Adults.

Among 5,530 records identifying patients who underwent CABG in the database of the BYPASS study, 2.589 presented data fulfilling inclusion criteria for analysis (Figure 1). Patients distributed according to BMI composed three groups: normal weight (767), overweight (1,146), and obesity (676). Table 1 resumes the anthropometric and clinical data of patients at the preoperative and intraoperative periods. The obesity group who underwent CABG presented lower mean age compared to normal weight and overweight groups (*P*=0.001).

**Table 1:** Anthropometric and clinical data of patients according to body mass index.

Variables	Total (n=2589)	Normal weight (n=767)	Overweight (n=1146)	Obesity (n=676)	*P*-value
Age (years)^a^	63.5±9.5	64.5±9.9	63.7±9.2	61.9±9.5^^*^$^	< 0.001
BMI (kg/m^2^)^a^	27.5±4.3	22.9±1.5	27.3±1.4	32.1±3.0^^*^$^	< 0.001
Female sex, n (%)^b^	743 (28.7)	208 (27.1)	308 (26.9)	227 (33.6)^^*^$^	0.005
Hypertension, n (%)^b^	2194 (84.7)	599 (78.1)	990 (86.4)	605 (89.5)^^*^$^	< 0.001
Dyslipidemia, n (%)^b^	1357 (52.4)	349 (45.5)	615 (53.7)	393 (58.1)^*^	< 0.001
Diabetes, n (%)^b^	1108 (42.8)	277 (36.1)	483 (42.1)	348 (51.5)^*^$	< 0.001
Active smoker, n (%)^b^	313 (12.1)	132 (17.2)	110 (9.6)	71 (10.5)^*^$	< 0.001
Ex-smoker, n (%)^b^	649 (28.7)	177 (28.1)	289 (28.1)	183 (30.4)	0.557
History of CVD, n (%)^b^	926 (35.8)	258 (33.6)	411 (35.9)	257 (38.0)	0.222
Heart failure, n (%)^b^	338 (13.1)	110 (14.3)	143 (12.5)	85 (12.6)	0.451
Previous MI, n (%)^b^	1036 (40.0)	323 (42.1)	445 (38.8)	268 (39.6)	0.348
Previous PCI, n (%)^b^	339 (13.1)	89 (11.6)	159 (13.9)	91 (13.5)	0.334
LVEF (%)^a^	58.2±12.8	56.0±13.6	58.8±12.6	59.8±11.8	< 0.001
Peripheral arterial disease, n (%)^b^	174 (6.7)	59 (7.7)	78 (6.8)	37 (5.5)	0.241
Previous stroke, n (%)^b^	112 (4.3)	32 (4.2)	52 (4.5)	28 (4.1)	0.894
Chronic kidney disease, n (%)^b^	117 (4.5)	39 (5.1)	49 (4.3)	29 (4.3)	0.668
Preoperative arrhythmias, n (%)^b^	153 (5.9)	49 (6.4)	64 (5.6)	40 (5.9)	0.766
Baseline clinics					
Serum creatinine (mg/dL)^a^	1.05±0.77	1.05±0.82	1.07±0.79	1.04±0.66	0.441
RBC (g/dL)^a^	12.3±5.34	12.0±3.3	12.4±5.5	12.5±6.8	0.096
Blood glucose level (mmol/L)^a^	102±77	94±74.2	104±77.5	109±80.9	0.001
Operative characteristics					
Number of grafts, n^c^	3 (2 - 4)	3 (2 - 4)	3 (2 - 4)	3 (2 - 4)	0.943
CPB use^a^	2261 (87.4)	665 (86.7)	999 (87.2)	597 (88.4)	0.597
CPB time (min)^a^	69 (52 - 90)	68 (51 - 89)	70 (55 - 91)	65 (50 - 88)	0.721
Occluding clamp^a^	206 (9.1)	54 (8.1)	96 (9.6)	56 (9.4)	0.565
Total	57 (27.7)	13 (24.1)	31 (32.3)	13 (23.2)	0.381
Partial	149 (72.3)	41 (75.9)	65 (67.7)	43 (76.8)	-
Cardioplegia use, n (%)^a^	2156 (95.4)	625 (94.0)	950 (95.1)	625 (94.0)^^*^^	0.017
Vasoactive drug during operation, n (%)^a^	1271 (49.1)	398 (51.9)	558 (48.7)	315 (46.7)	0.132
Intraoperative mortality, n (%)^a^	7 (0.3)	1 (0.1)	4 (0.3)	2 (0.3)	0.658

Numerical data are presented as mean ± standard deviation or as median (25-75% percentiles), categorical data are presented as absolute (relative) frequencies, analyzed with aone-way analysis of variance for independent samples and

b χ^2^ test, respectively

c Kruskal-Wallis test ^*^P<0.03 for pairwise comparison with normal weight

$ *P*<0.03 for pairwise comparison with overweight

BMI=body mass index; CPB=cardiopulmonary bypass; CVD=cardiovascular disease; LVEF=left ventricular ejection fraction; MI=myocardial infarction; PCI=percutaneous coronary intervention; RBC=red blood cells count

The distribution of age, sex, diabetes, and dyslipidemia significantly varied among groups. The female sex was more prevalent in the obesity group compared to both normal weight (*P*=0.02) and overweight groups (*P*=0.007). Patients in the overweight and obesity groups had a higher prevalence of diabetes in the preoperative period compared to the normal weight group (*P*=0.025 and *P*<0.001, respectively) (Table 1). Also, diabetes was higher in obese people compared to the overweight group (*P*<0.001). A higher number of patients with hypertension was observed in the overweight and obesity groups compared to the normal weight group in the preoperative period (*P*<0.001) (Table 1), and no significant difference in hypertension prevalence was observed between obesity and overweight groups. Finally, dyslipidemia was more frequently observed in the overweight and obesity groups compared to normal weight group in the preoperative period (*P*<0.001 and *P*<0.002, respectively) (Table 1).

### Clinical Outcomes During the Inpatient Period

Since patients presented significant differences in the baseline, analyses of clinical outcomes were adjusted for age, sex, hypertension, and diabetes to deal with possible confounders. While comparing the three groups (normal weight, obesity, overweight), it was not observed significant differences in prolonged ICU stay (χ^2^ = 0.36, *P*=0.836) (Table 2), prolonged hospitalization (χ^2^ = 1.74, *P*=0.41), and mortality rate (χ^2^ = 0.67, *P*=0.714) during inpatient period (Table 2). SWI rate was higher in the obesity group compared to normal weight (relative risk [RR] = 5.89, 95% confidence interval [CI] = 2.37 - 17.82; *P*=0.001) (Table 3 and Table S1). Age was an independent predictor of prolonged MV, acute respiratory distress syndrome, stroke, renal failure, new arrhythmias, and prolonged hospitalization (Table S1). Female sex was independently associated with prolonged hospitalization and in-hospital mortality (RR = 2.79, 95% CI = 1.64 - 4.79; *P*<0.001) (4.2% *vs.* 1.5%, χ^2^ = 17.8, *P*<0.001) (Table S1).

**Table 2:** Postoperative clinical outcomes during inpatient, 30-day, six-month, and one-year follow-up periods according to body mass index groups.

Inpatient outcomes	Total (n=2589)	Normal weight (n=767)	Overweight (n=1146)	Obesity (n=676)	*P*-value
Prolonged MV, n (%)	580 (22.4)	170 (22.2)	253 (22.1)	157 (23.2)	0.836
ARDS, n (%)	34 (1.3)	9 (1.2)	18 (1.6)	7 (1.0)	0.576
LCOS, n (%)	78 (3.0)	24 (3.1)	38 (3.3)	16 (2.4)	0.506
Renal failure, n (%)	88 (3.4)	24 (3.1)	40 (3.5)	24 (3.6)	0.884
Stroke, n (%)	31 (1.2)	10 (1.3)	12 (1.0)	9 (1.3)	0.821
Need for insulin, n (%)	513 (19.8)	134 (17.5)	222 (19.4)	157 (23.2)	**0.021**
New arrhythmias, n (%)	431 (16.6)	121 (15.8)	196 (17.1)	114 (16.9)	0.736
Bleeding, n (%)	76 (2.9)	32 (4.2)	32 (2.8)	12 (1.8)^*^	**0.025**
Early reoperation, n (%)	60 (2.3)	18 (2.3)	29 (2.5)	13 (1.9)	0.706
SWI, n (%)	119 (4.6)	11 (1.4)	49 (4.3)	59 (8.8)^*^	**< 0.001**
Prolonged ICU stay, n (%)	580 (22.4)	170 (22.2)	253 (22.1)	157 (23.2)	0.836
Prolonged hospitalization, n (%)	128 (4.9)	36 (4.7)	51 (4.5)	41 (6.0)	0.294
Hospital mortality, n (%)	58 (2.2)	20 (2.6)	24 (2.1)	14 (2.1)	0.714
**30-day outcomes**	**Total**	**Normal weight**	**Overweight**	**Obesity**	***P*-value**
	(n=2142)	(n=641)	(n=931)	(n=570)	
Renal failure, n (%)	26 (1.2)	5 (0.8)	10 (1.1)	11 (1.9)	0.166
Stroke, n (%)	13 (0.6)	4 (0.6)	3 (0.3)	6 (1.1)	0.209
Heart Failure, n (%)	16 (0.7)	4 (0.6)	5 (0.5)	7 (1.2)	0.288
30-day rehospitalization, n (%)	113 (5.3)	35 (5.5)	42 (4.5)	36 (6.3)	0.302
30-day mortality, n (%)	26 (1.1)	6 (0.9)	12 (1.3)	8 (1.4)	0.745
**6-month outcomes**	**Total**	**Normal weight**	**Overweight**	**Obesity**	***P*-value**
	(n=1635)	(n=499)	(n=716)	(n=420)	
Renal failure, n (%)	15 (0.9)	2 (0.4)	6 (0.8)	7 (1.7)	0.126
Stroke, n (%)	6 (0.4)	1 (0.2)	3 (0.4)	2 (0.5)	0.752
Heart failure, n (%)	10 (0.6)	2 (0.4)	6 (0.8)	2 (0.5)	0.578
6-month rehospitalization, n (%)	92 (5.6)	18 (3.6)	44 (6.2)	30 (7.1)^*^	**0.040**
Surgical intervention at 6-month, n (%)	30 (1.8)	4 (0.8)	12 (1.7)	14 (3.3)^*^	**0.016**
6-month mortality, n (%)	20 (1.2)	7 (1.4)	9 (1.3)	4 (0.9)	0.811
**1-year outcomes**	**Total**	**Normal weight**	**Overweight**	**Obesity**	***P*-value**
	(n=1049)	(n=318)	(n=462)	(n=269)	
Renal failure, n (%)	2 (0.2)	0 (0.0)	2 (0.4)	0 (0.0)	0.280
Stroke, n (%)	2 (0.2)	0 (0.0)	2 (0.4)	0 (0.0)	0.278
Heart failure, n (%)	10 (1.0)	2 (0.6)	4 (0.9)	4 (1.5)	0.555
1-year rehospitalization, n (%)	29 (2.8)	8 (2.5)	16 (3.5)	5 (1.9)	0.420
1-year mortality, n (%)	7 (0.7)	0 (0.0)	5 (1.1)	2 (0.7)	0.188

Data are presented as absolute (relative) frequencies and analyzed with χ^^2^^ test

* *P*<0.05 for pairwise comparison of obesity *vs.* normal weight

ARDS=acute respiratory distress syndrome; ICU=intensive care unit; LCOS=low cardiac output syndrome; MV=mechanical ventilation; SWI=sternal wound infections

**Table 3:** Generalized linear models (GzLM) with logistic distribution for outcome variables exploring obesity group compared to normal weight group.

Variables	RR	95% CI	z	*P*-value
Prolonged MV	1.05	0.65 - 1.68	0.208	0.835
ARDS	1.05	0.37 - 2.91	0.104	0.917
LCOS	0.78	0.40 - 1.48	-0.760	0.447
Renal failure	1.13	0.63 - 2.04	0.418	0.676
Stroke	1.06	0.41 - 2.71	0.132	0.895
Need for insulin	1.08	0.81 - 1.43	0.537	0.932
New arrhythmias	1.26	0.94 - 1.69	1.579	0.114
Bleeding	0.44	0.21 - 0.85	-2.320	0.061
Early reoperation	0.89	0.42 - 1.87	-0.290	0.772
SWI	5.89	2.37 - 17.82	3.51	**0.001**
Prolonged ICU stay	0.88	0.68 - 1.14	-0.971	0.332
Prolonged hospitalization	1.25	0.74 - 2.10	0.822	0.411
Inpatient mortality	0.72	0.35 - 1.46	-0.892	0.372
30-day rehospitalization	1.21	0.72 - 2.03	0.725	0.469
30-day mortality	2.19	0.73 - 6.97	1.394	0.163
6-month rehospitalization	2.16	1.17 - 4.09	2.433	**0.045**
Need for surgical intervention	4.38	1.53 - 15.77	2.546	**0.033**
6-month mortality	0.62	0.16 - 2.13	-0.739	0.460
1-year rehospitalization	0.69	0.21 - 2.16	-0.614	0.539

GzLM with logistic distribution for dependent variables, adjusted for age, sex, hypertension, and diabetes ARDS=acute respiratory distress syndrome; CI=confidence interval; ICU=intensive care unit; LCOS=low cardiac output syndrome; MV=mechanical ventilation; RR=relative risk; SWI=sternal wound infections

**Table S1:** Generalized linear models (GzLM) with logistic distribution for outcome variables during inpatient postoperative period.

Variables	RR	95% CI	z	*P*-value
**Prolonged MV**				
Obesity - normal weight	1.05	0.65 - 1.68	0.208	0.835
Overweight - normal weight	0.93	0.61 - 1.40	-0.367	0.713
Age	1.04	1.02 - 1.07	4.440	**< 0.0001**
Sex (female - male)	1.32	0.91 - 1.91	1.482	0.138
Diabetes	0.93	0.64 - 1.32	-0.410	0.681
Hypertension	0.89	0.56 - 1.50	-0.434	0.664
**ARDS**				
Obesity - normal weight	1.05	0.37 - 2.91	0.104	0.917
Overweight - normal weight	1.47	0.66 - 3.48	0.923	0.356
Age	1.09	1.05 - 1.14	4.374	**< 0.001**
Sex (female - male)	1.92	0.95 - 3.84	1.845	0.065
Diabetes	0.75	0.36 - 1.50	-0.810	0.418
Hypertension	1.58	0.55 - 6.71	0.744	0.457
**LCOS**				
Obesity - normal weight	0.78	0.40 - 1.48	-0.760	0.447
Overweight - normal weight	1.05	0.63 - 1.81	0.206	0.837
Age	1.02	0.99 - 1.05	1.830	0.067
Sex (female - male)	1.38	0.84 - 2.22	1.314	0.189
Diabetes	0.99	0.61 - 1.57	-0.053	0.958
Hypertension	0.93	0.50 - 1.84	-0.242	0.808
**Renal failure**				
Obesity - normal weight	1.13	0.63 - 2.04	0.418	0.676
Overweight - normal weight	1.06	0.64 - 1.82	0.249	0.803
Age	1.03	1.01 - 1.06	2.541	**0.011**
Sex (female - male)	0.94	0.57 - 1.49	-0.270	0.787
Diabetes	1.25	0.81 - 1.93	0.997	0.319
Hypertension	1.63	0.82 - 3.72	1.281	0.200
**Stroke**				
Obesity - normal weight	1.06	0.41 - 2.71	0.132	0.895
Overweight - normal weight	0.82	0.35 - 1.95	-0.468	0.639
Age	1.06	1.02 - 1.11	2.996	**0.003**
Sex (female - male)	1.65	0.78 - 3.39	1.343	0.179
Diabetes	1.31	0.63 - 2.72	0.726	0.468
Hypertension	1.38	0.47 - 5.88	0.524	0.600
**Need for insulin**				
Obesity - normal weight	1.08	0.81 - 1.43	0.537	0.932
Overweight - normal weight	1.01	0.78 - 1.31	0.085	0.591
Age	1.00	0.99 - 1.02	0.738	0.461
Sex (female - male)	1.17	0.93 - 1.46	1.355	0.175
Diabetes	7.49	5.93 - 9.54	16.603	**< 0.001**
Hypertension	0.89	0.65 - 1.24	-0.667	0.505
**New arrhythmias**				
Obesity - normal weight	1.26	0.94 - 1.69	1.579	0.114
Overweight - normal weight	1.15	0.89 - 1.49	1.074	0.283
Age	1.06	1.04 - 1.07	8.914	**< 0.001**
Sex (female - male)	0.92	0.72 - 1.17	-0.680	0.497
Diabetes	0.88	0.71 - 1.09	-1.127	0.260
Hypertension	1.28	0.93 - 1.78	1.466	0.143
**Bleeding**				
Obesity - normal weight	0.44	0.21 - 0.85	-2.320	0.061
Overweight - normal weight	0.66	0.39 - 1.09	-1.604	0.109
Age	1.01	0.99 - 1.05	1.176	0.239
Sex (female - male)	0.69	0.38 - 1.19	-1.258	0.208
Diabetes	0.98	0.60 - 1.58	-0.066	0.947
Hypertension	1.15	0.62 - 2.36	0.428	0.668
**Reoperation**				
Obesity - normal weight	0.89	0.42 - 1.87	-0.290	0.772
Overweight - normal weight	1.15	0.63 - 2.16	0.450	0.653
Age	1.02	0.99 - 1.05	1.524	0.127
Sex (female - male)	0.68	0.35 - 1.24	-1.187	0.235
Diabetes	1.26	0.74 - 2.14	0.868	0.385
Hypertension	0.95	0.48 - 2.12	-0.119	0.906
**SWI**				
Obesity - normal weight	5.89	2.37 - 17.82	3.51	**0.001**
Overweight - normal weight	2.96	1.19 - 8.98	2.153	0.094
Age	0.97	0.96 - 1.03	-1.425	0.887
Sex (female - male)	1.08	0.56 - 1.98	0.240	0.810
Diabetes	1.85	1.03 - 3.39	2.02	**0.043**
Hypertension	0.99	0.44 - 2.69	-0.003	0.997
**Prolonged ICU stay**				
Obesity - normal weight	0.88	0.68 - 1.14	-0.971	0.332
Overweight - normal weight	0.98	0.78 - 1.22	-0.199	0.842
Age	0.99	0.97 - 1.00	-2.490	0.013
Sex (female - male)	1.07	0.87 - 1.33	0.700	0.484
Diabetes	1.06	0.88 - 1.29	0.647	0.518
Hypertension	1.32	1.03 - 1.69	2.176	**0.030**
**Prolonged hospitalization**				
Obesity - normal weight	1.25	0.74 - 2.10	0.822	0.411
Overweight - normal weight	0.95	0.58 - 1.55	-0.218	0.827
Age	1.05	1.02 - 1.07	3.941	**< 0.001**
Sex (female - male)	2.06	1.37 - 3.07	3.531	**< 0.001**
Diabetes	1.79	1.19 - 2.72	2.809	**0.005**
Hypertension	1.09	0.61 - 2.16	0.291	0.771
**Hospital mortality**				
Obesity - normal weight	0.72	0.35 - 1.46	-0.892	0.372
Overweight - normal weight	0.75	0.41 - 1.39	-0.913	0.361
Age	1.02	0.99 - 1.05	1.249	0.212
Sex (female - male)	2.79	1.64 - 4.79	3.771	**< 0.001**
Diabetes	1.06	0.61 - 1.81	0.210	0.834
Hypertension	1.37	0.62 - 3.65	0.716	0.474

ARDS=acute respiratory distress syndrome; CI=confidence interval; ICU=intensive care unit; LCOS=low cardiac output syndrome; MV=mechanical ventilation; RR=relative risk; SWI=sternal wound infections

### Clinical Outcomes in the 30-Day Follow-up

Rehospitalization rates were similar among the three groups during the 30-day follow-up period (χ^2^ = 2.03, *P*=0.363) (Table 2). Similarly, mortality rates did not differ significantly among groups (χ^2^ = 0.66, *P*=0.721). Age was independently associated with rehospitalization and mortality within 30 days after discharge (Table S1). Sex and diabetes were determinants to rehospitalization within 30 days, female sex expressed a 1.5-fold risk (RR = 1.53, 95% CI = 1.00 - 2.31; *P*=0.045) (Table S2), while diabetes represented a 1.7-fold increase in the risk for rehospitalization (RR = 1.73, 95% CI = 1.15 - 2.62; *P*=0.008) (Table 3 and Table S2).

**Table S2:** Generalized linear models (GzLM) with logistic distribution for outcome variables.

Variables	RR	95% CI	z	*P*-value
**30-day follow-up**				
*Rehospitalization*				
Obesity - normal weight	1.21	0.72 - 2.03	0.725	0.469
Overweight - normal weight	0.84	0.52 - 1.37	-0.698	0.485
Age	1.03	1.01 - 1.05	2.673	**0.008**
Sex (female - male)	1.53	1.00 - 2.31	2.008	**0.045**
Diabetes	1.73	1.15 - 2.62	2.633	**0.008**
Hypertension	0.68	0.40 - 1.17	-1.457	0.145
*30-day mortality*				
Obesity - normal weight	2.19	0.73 - 6.97	1.394	0.163
Overweight - normal weight	1.64	0.61 - 4.83	0.963	0.336
Age	1.16	1.10 - 1.22	5.404	**< 0.001**
Sex (female - male)	0.79	0.30 - 1.87	-0.495	0.620
Diabetes	1.06	0.47 - 2.39	0.154	0.877
Hypertension	1.22	0.40 - 5.28	0.312	0.755
**6-month follow-up**				
*Rehospitalization*				
Obesity - normal weight	2.16	1.17 - 4.09	2.433	**0.045**
Overweight - normal weight	1.87	1.07 - 3.41	2.126	0.100
Age	1.01	0.98 - 1.03	0.660	0.509
Sex (female - male)	1.19	0.75 - 1.89	0.771	0.441
Diabetes	1.08	0.69 - 1.67	0.340	0.733
Hypertension	1.02	0.57 - 1.97	0.066	0.947
*Need for surgical intervention*				
Obesity - normal weight	4.38	1.53 - 15.77	2.546	**0.033**
Overweight - normal weight	2.11	0.73 - 7.61	1.277	0.202
Age	1.04	1.00 - 1.08	1.970	0.049
Sex (female - male)	1.36	0.61 - 2.87	0.791	0.429
Diabetes	1.05	0.49 - 2.19	0.116	0.907
Hypertension	2.08	0.61 - 13.09	0.991	0.322
*6-month mortality*				
Obesity - normal weight	0.62	0.16 - 2.13	-0.739	0.460
Overweight - normal weight	0.82	0.29 - 2.33	-0.391	0.695
Age	1.09	1.04 - 1.16	3.141	**0.002**
Sex (female - male)	1.66	0.65 - 4.11	1.098	0.272
Diabetes	4.86	1.73 - 17.32	2.764	**0.006**
Hypertension	2.54	0.51 - 46.35	0.898	0.369
**1-year follow-up**				
*Rehospitalization*				
Obesity - normal weight	0.69	0.21 - 2.16	-0.614	0.539
Overweight - normal weight	1.50	0.64 - 3.80	0.906	0.365
Age	0.98	0.94 - 1.02	-0.955	0.339
Sex (female - male)	2.65	1.23 - 5.68	2.531	**0.011**
Diabetes	2.05	0.95 - 4.57	1.800	0.072
Hypertension	0.42	0.18 - 1.04	-1.999	0.046

GzLM with logistic distribution for dependent variables, adjusted for age, sex, hypertension, and diabetes during 30-day, six-month, and one-year follow-up CI=confidence interval; RR=relative risk

### Clinical Outcomes in the Six-Month Follow-up

Rehospitalization rates in six months after discharge were higher in the obesity and overweight groups than in normal-weight patients (7.1% and 6.2% *vs.* 3.6%, respectively, χ^2^ = 6.03; *P*=0.049) (Table 2). Obese patients conveyed a 2.2-fold increase in the risk for rehospitalization within six months after discharge compared to normal-weight patients (RR = 2.16, 95% CI = 1.17 - 4.09; *P*=0.045) (Table 3 and Table S2), adjusted for age, sex, hypertension, and diabetes. Also, obese patients presented a higher rate of need for surgical intervention within six months after discharge compared to normal-weight patients (3.3% *vs.* 0.8%, χ^2^ = 8.29; *P*=0.016). Age and diabetes were significantly and independently associated with 6-month mortality rates (Table S2). Diabetes was related to a 4.8-fold increase in the odds for mortality rate within six months (RR = 4.86, 95% CI = 1.73 - 17.32; *P*=0.006) (Table 3 and Table S2).

### Clinical Outcomes in the One-Year Follow-up

Rehospitalization and mortality rates did not differ among the three groups in the one-year follow-up. Female sex was independently associated with rehospitalization in one year following discharge compared to male sex (RR = 2.65, 95% CI = 1.23 - 5.68; *P*=0.011) (Table 3 and Table S2). There was not enough data regarding deaths within one year after discharge to enable performing GzLM analysis adjusted for confounders. Diabetic patients presented a higher rate of deaths within one year than those without this morbidity (1.5% *vs.* 0%, respectively, χ^2^ = 9.11; Fisher’s exact test *P*=0.003).

## DISCUSSION

Our findings revealed that obese and overweight patients had higher rates of diabetes, hypertension, and dyslipidemia as baseline conditions before CABG. However, no statistically significant differences were observed in clinical outcomes during hospitalization, except for higher SWI rates in obese compared to eutrophic individuals. During postoperative follow-up, obese patients had higher rates of surgical reintervention and rehospitalization within six months after CABG, even adjusting for comorbidities. As a result, findings from the present study raise doubts about the obesity paradoxical effect on patients’ hospital mortality following surgery, when comparing adjusted data of normal weight and overweight groups.

Obesity has grown epidemic worldwide, especially in low and medium-income countries, like Brazil^[21]^. The increase in this condition is a challenge for public health, since obesity is a risk factor for other chronic diseases, such as metabolic syndrome and CAD[18].

Discrepancies around obesity associations with clinical results are frequently debated in the literature. Although obesity may represent a high risk for cardiovascular disease and metabolic syndrome, its presence may be protective during the postoperative clinical course of cardiac surgery. In this context, cardiac surgery is still considered a safe approach, even in higher-risk populations^[22]^. However, previous data on the effects of obesity on clinical outcomes and postoperative mortality are controversial^[22,23]^. To our knowledge, this is the first multicenter prospective study performed with the Brazilian population analyzing the effect of BMI and obesity as an independent predictor of clinical outcomes after CABG.

The current study revealed that obese and overweight patients had higher rates of hypertension compared to normal-weight patients. These data corroborate recent findings from a meta-analysis that suggest that increased cardiac output is the main cause of hypertension in young adults, a condition frequently associated with obesity^[24]^. One of the mechanisms described to explain this association is the increase in sympathetic activation found in obese patients, observed by recording muscle sympathetic nerve activation^[25]^, as well as an increase in cardiac output^[26]^. Therefore, hypertension is a very common comorbidity in obese patients, increasing the risk of cardiac events that may lead to the need for surgery.

Diabetes is one of the main preoperative risk factors found in patients undergoing cardiovascular interventions and has been significantly growing in the Brazilian population. Data from the National Health Survey report a 35% increase in diabetes incidence in 2019 compared to 2013 data^[27]^. The prevalence of diabetes in obese individuals is widely reported, authors describe an increase in insulin resistance and glucose intolerance^[28]^. In fact, this analysis of the BYPASS Registry database revealed that obese and overweight individuals presented higher rates of diabetes compared to eutrophic people. This data is compatible with previous reports on obese patients undergoing cardiac surgery, all studies found higher rates of hypertension, diabetes, dyslipidemia, heart failure, and other comorbidities during the preoperative evaluation^[7,22,23]^. Since comorbidities may be associated with worse outcomes, it is important to attempt to isolate the effect of comorbidities from the presence of obesity and overweight during analyses. Therefore, the present study investigated the association of BMI groups on CABG results taking into account the presence of hypertension and diabetes, two of the most frequent and influential baseline comorbidities.

The estimates of the effects varied among the studies depending on the types of surgeries considered, hence only patients undergoing CABG were included in the analysis of the current study. Moreover, our study excluded COPD patients from all groups to exclude the presence of a potential confounder, since COPD is described as an independent factor for postoperative complications and mortality^[29]^. However, Johnson et al.^[7]^ reported that even with a higher rate of COPD in the obesity group, which would impact the European System for Cardiac Operative Risk Evaluation (EuroSCORE) II, a controversial lower mortality rate was observed. It is noteworthy that the EuroSCORE II does not consider BMI into risk calculation, as the correlation between BMI and mortality risk was found to be minimal during the development of the model. This suggests that weight alone is not a major determinant of outcomes when underlying weight-related conditions such as diabetes mellitus and renal dysfunction are accounted for.

Recent findings report a possible cardioprotective role related to obesity, this phenomenon was named the obesity paradox. Studies indicate that obesity reduces the risk of mortality in patients undergoing cardiac surgery or who are diagnosed with heart failure. The reason for this phenomenon is tied to symptoms and hypertension. These patients may be operated on early due to the faster presence of dyspnea and lower limb edema, while combining obesity and heart disease. They are able to tolerate higher doses of cardioprotective medications such as betablockers due to higher blood pressure levels, which helps them to maintain preserved renal function. Obesity is related to higher serum levels of lipoproteins and adipokines such as tumor necrosis factor-alpha, which would somehow neutralize inflammatory components^[30-32]^. Another explanation is the higher percentage of lean mass in obese individuals compared to eutrophic patients with cardiovascular diseases such as heart failure, which would bring them the advantage of better cardiorespiratory fitness^[33]^. On the other hand, other studies refuted these findings, stating that the heterogeneity of the sample would be a confounding factor for the results.

In a systematic review, Mariscalco et al.^[21]^ suggest the presence of selection bias, where obese patients with more severe heart diseases, which would make surgical interventions riskier, were excluded from the studies, so there would be no parity in surgical risk between groups. These assumptions reveal that obese patients with higher risk may not be referred for cardiac surgery and may not even be included in these studies. Additionally, studies about the obesity paradox presented an extensive number of samples; some studies included 78 to 350 thousand patients. It is important to notice that such large samples can evolve with type 2 error of statistics. Upon close examination of the data, studies showed that obese and overweight patients had a lower risk of mortality, but the difference from normal-weight individuals did not result in a reduction of mortality by even 2%. Controversial to these previous studies, our findings do not reveal lower mortality following CABG in obese patients.

The current investigation around BYPASS Registry database revealed similar rates of mortality during inpatient period and in the one-year follow-up. Only age, presence of diabetes, and female sex were independently associated with prolonged hospitalization and mortality. Obesity was found to be an independent predictor for SWI. The present results revealed a 5.89 higher risk of SWI in obese patients compared to normal-weight patients. Several studies confirm this finding, with a similar previously reported risk of 1.3 to 6.9 to evolve with this outcome^[34,35]^. Finally, among all clinical outcomes investigated in this study, obese patients presented a higher risk of six-month rehospitalization. We believed that SWI may play an important role in this outcome, since the need for surgical intervention was also observed in this period. To the best of our knowledge, this finding has not been well explored in the literature. The risk of hospitalization in the mid-term can guide more effective clinical follow-up strategies aimed at improving the quality of life and reducing costs during treatment after surgery of obese patients.

Given that obesity itself was no longer associated with reduced risk of mortality, the investigation around physical fitness during the preoperative should be more complex than observing BMI. Despite the fact that obesity was associated with SWI and six-month rehospitalization, this classification may not be useful to assess risk during the perioperative period. Studies have been discussing the status of body composition that would define different fitness categories. It has been described that some obese patients presented a larger lean body mass, *i.e.*, metabolically healthy obese, that would perform better than eutrophics considered as metabolically obese normal-weight patients, in other words, latent obesity. Moreover, a sarcopenic obese would evolve with the worst outcome among all types of body composition^[36,37]^.

The best investigation of physical fitness would include a more robust and dynamic evaluation, such as exercise tolerance. Rocco et al.^[38]^ found that patients with delayed capacity of oxygen consumption during a walking test presented a higher risk of postoperative complications, which consolidates the theory around body composition and dynamic fitness over BMI.

### Limitations

The data collected by the BYPASS project represents the experiences of a select group of hospitals across the country who voluntarily participated and provided the required information through a dedicated questionnaire. These participating hospitals may not accurately reflect the national standard, and a registry with a larger number of institutions would help to address this concern. Nevertheless, the data obtained from these hospitals provide clinical data about obesity’s role as a predictor of postoperative outcomes.

## CONCLUSION

Obesity increased the risk for SWI, leading to higher rehospitalization rates and need for surgical interventions within six months following CABG. Only age, female sex, and diabetes were associated with a higher risk of worse clinical outcomes and mortality. The obesity paradox remains controversial since BMI may not be sufficient to assess postoperative risk in light of more complex and dynamic evaluations of body composition and physical fitness.
